# Comprehensive genomic and tumour immune profiling reveals potential therapeutic targets in malignant pleural mesothelioma

**DOI:** 10.1186/s13073-022-01060-8

**Published:** 2022-05-30

**Authors:** Jenette Creaney, Ann-Marie Patch, Venkateswar Addala, Sophie A. Sneddon, Katia Nones, Ian M. Dick, Y. C. Gary Lee, Felicity Newell, Ebony J. Rouse, Marjan M. Naeini, Olga Kondrashova, Vanessa Lakis, Apostolos Nakas, David Waller, Annabel Sharkey, Pamela Mukhopadhyay, Stephen H. Kazakoff, Lambros T. Koufariotis, Aimee L. Davidson, Priya Ramarao-Milne, Oliver Holmes, Qinying Xu, Conrad Leonard, Scott Wood, Sean M. Grimmond, Raphael Bueno, Dean A. Fennell, John V. Pearson, Bruce W. Robinson, Nicola Waddell

**Affiliations:** 1 National Centre for Asbestos Related Disease, Medical School, University of Western Australia, Level 5, QQ Block, QEII Medical Centre, 6 Verdun Street, Nedlands, WA 6009 Australia; 2 Department of Respiratory Medicine, Sir Charles Gairdner Hospital, Nedlands, WA Australia; 3 Centre for Respiratory Health, University of Western Australia, Nedlands, WA Australia; 4 Medical Genomics, Clinical Genomics and Genome Informatics Groups, QIMR Berghofer Medical Research Institute, 300 Herston Road, Herston, Brisbane, QLD 4006 Australia; 5 Faculty of Medicine, The University of Queensland, Brisbane, QLD Australia; 6 Cancer Research UK Centre Leicester, University of Leicester & University Hospitals of Leicester NHS Trust, Leicester, UK; 7 University of Melbourne Centre for Cancer Research, University of Melbourne, Melbourne, VIC Australia; 8 Division of Thoracic Surgery, Brigham and Women’s Hospital, Boston, MA USA

**Keywords:** Malignant pleural mesothelioma, Whole genome sequencing, RNA sequencing, Mutational signatures, Tumour micro-environment, Immunotherapy

## Abstract

**Background:**

Malignant pleural mesothelioma (MPM) has a poor overall survival with few treatment options. Whole genome sequencing (WGS) combined with the immune features of MPM offers the prospect of identifying changes that could inform future clinical trials.

**Methods:**

We analysed somatic mutations from 229 MPM samples, including previously published data and 58 samples that had undergone WGS within this study. This was combined with RNA-seq analysis to characterize the tumour immune environment.

**Results:**

The comprehensive genome analysis identified 12 driver genes, including new candidate genes. Whole genome doubling was a frequent event that correlated with shorter survival. Mutational signature analysis revealed SBS5/40 were dominant in 93% of samples, and defects in homologous recombination repair were infrequent in our cohort. The tumour immune environment contained high M2 macrophage infiltrate linked with *MMP2*, *MMP14*, *TGFB1* and *CCL2* expression, representing an immune suppressive environment. The expression of *TGFB1* was associated with overall survival. A small subset of samples (less than 10%) had a higher proportion of CD8 T cells and a high cytolytic score, suggesting a ‘hot’ immune environment independent of the somatic mutations.

**Conclusions:**

We propose accounting for genomic and immune microenvironment status may influence therapeutic planning in the future.

**Supplementary Information:**

The online version contains supplementary material available at 10.1186/s13073-022-01060-8.

## Background

Malignant pleural mesothelioma (MPM) is a rapidly lethal cancer of the mesothelial lining involving the pleura that is causally linked with asbestos exposure [1, 2]. Genomics studies of MPM have focused mostly on exome sequencing in combination with copy number, gene expression or methylation analysis [3–5]. These studies have confirmed that MPM is an unusual cancer predominantly driven by loss of tumour suppressors (*BAP1*, *NF2* and *CDKN2A*) with a lack of oncogenic gain-of-function events. Treatment options, including chemotherapy, radiotherapy and surgery, remain largely ineffectual. Genome-guided medicine has yielded benefits in other cancer types, but the translation of genomic knowledge for MPM is yet to be realized.

Targeted HDAC, EZH2 and PARP treatments are being pursued based upon *BAP1* status (clinical trial: NCT03207347) and its purported role in chromatin remodelling, transcriptional regulation and DNA repair, respectively [6]. In addition, recent combination immunotherapy trial results offer new treatment options [7–9]. With the FDA recently approving combination nivolumab and ipilimumab treatment for patients with unresectable MPM [7], a treatment that also induces good responses in patients with the historically intractable sarcomatoid histology. Interestingly, MPM lacks the genomic markers that have been generally associated with immunotherapy responsiveness, such as, high tumour mutation burden [10–12], high neoantigen load and mutational signatures associated with mismatch repair or homologous recombination repair deficiency [13]. Therefore, a deep genomic or immunological characterization of MPM samples may reveal alternative features that could influence treatment choices.

We performed WGS on 58 MPM tumours with matched transcriptome sequencing and combined this with publicly available data to provide a cohort of over 200 MPMs [3, 4]. This enabled an in-depth characterization of mutational signatures and driver genes, plus an exploration of the tumour immune microenvironment.

## Methods

### Clinical cohort description and sample processing

We profiled 58 pathologically confirmed MPM tumour samples and matched germline DNA from patients with MPM; the data from the 58 MPM samples is referred to as the ‘Creaney et al.’ data throughout the manuscript. The source, sample type and associated clinical details relating to the 58 MPM patients are in Additional file 1: Table S1. The 58 MPM samples were comprised of pleura tissue samples (*n* = 21), pleural effusions (*n* = 29) and low passage pleural effusion cell lines (*n* = 8). Samples were collected for genome-based studies with written informed consent from participants and approval of the Dana-Farber Brigham and Women’s Cancer Center Institutional Review Board (Boston, MA, USA) (*n* = 4 patients), University Hospitals of Leicester National Health Service Trust (Leicester, UK) (*n* = 12 patients) and the Sir Charles Gairdner and Osborne Park Hospital Research Ethics Committee (SCGOPH REC; Perth, Western Australia, Australia) (*n* = 42 patients). Pleural tumour tissue was snap frozen in liquid nitrogen after surgical removal. Pleural effusions were drained as clinical indicated then transported to the laboratory at room temperature for processing. For 29 samples, tumour cells were enriched directly from the pleural effusions by CD45 depletion following the manufactures directions (Stemcell Technologies, Tullamarine, Victoria, Australia). For 8 samples, an aliquot of the effusion cell pellet was placed into RPMI-1640 media (Gibco) supplemented with 15% foetal calf serum, 200 mM Hepes, 10 mM 2-mercaptoethanol, 1x glutamax (Gibco), 1x nonessential amino acids (Gibco), plus 1x sodium pyruvate (Gibco) and tumour cells enriched through culturing at 37^o^C in a 5%CO_2_ humidified atmosphere for between 4 and 10 passages as previously described [14]. All cultures were confirmed to be Mycoplasma spp. free by polymerase chain reaction. Nucleic acids were extracted from pleura tissue, effusion, low passage effusion and blood samples using the Qiagen AllPrep Universal kit. MPM and matched normal DNA samples were screened using an Illumina SNP array and tumour purity assessed using the qpure tool [15]; samples with > 40% tumour cell content were selected for WGS. The WGS analysis was conducted at QIMR Berghofer with approval from the QIMR Berghofer Research Ethics Committee (P3521). Approval for this study was granted by SCGOPH REC (RGS0000001517).

### Whole genome sequencing generation and processing

Whole genome sequencing was performed on 58 MPM and matched germline DNA samples. Sequence libraries were generated from 500 ng DNA using the Illumina TruSeq DNA PCR-free (350 bp insert) kit and sequenced using paired-end sequencing reads of 150 bp with an HiSeq X Ten (Illumina, San Diego, CA, USA) at The Kinghorn Cancer Centre, Garvan Institute of Medical Research (Sydney, Australia) or Macrogen (Seoul, South Korea). Sequence reads were trimmed using Cutadapt [16] (v1.11) and aligned to the GRCh37 human reference using BWA-MEM [17] (v0.7.12). Duplicate alignments were marked with Picard (version 1.129, http://picard.sourceforge.net), and BAM files were coordinate-sorted using Samtools [18] (v1.3). Mean coverage was determined using qcoverage [19].

### Somatic variant calling

Single nucleotide variants (SNV) were detected using a dual calling strategy using qSNP [20] and the GATK HaplotypeCaller [21] and indels (1–50 bp) were called with the GATK HaplotypeCaller as previously described [22]. Variants were annotated with the Ensembl v75 gene feature information and transcript or protein consequences using SnpEff (version 4.2). Structural variants were determined using qSV as previously described [23] and annotated for their potential consequence on genes. Positions of breakpoints were annotated using the Ensembl v75 gene model. Copy number was determined using ascatNGS [24]. Samples identified as having undergone a whole genome duplication event had an overall ploidy of > 2.7 and > 50% of the genome estimated as copy number 3 to 6 with two alleles present, i.e. heterozygosity of those segments with copy number gain. We grouped copy number alterations to high gain (copy number ≥ 6) and homozygous deletion (copy number 0). Recurrent copy number changes were detected for chromosomal regions using GISTIC2.0 [25]. Significant genes with focal copy number changes were identified using confidence level of 0.95 and a *q*-value < 0.05.

### Driver gene analysis

Driver genes were identified based on the frequency and functional impact bias of variants. We applied a consensus approach using three tools to detect significantly mutated genes (SMG) affected by single nucleotide variants and indels. We performed SMG meta-analysis by merging Mutation Annotation Format (MAF) files of the samples from three studies: this study (Creaney et al.), TCGA [4] and Bueno et al. [3]. The SMG methods, including OncodriveFML [26], MutPanning [27] (V2.0) and MutSigCV [28], were run using default parameters. OncodriveFML was executed using CADD v1.0 via the web interface (http://bbglab.irbbarcelona.org/oncodrivefml/home). MutSigCV and MutPanning were executed using modules in GenePattern (https://www.genepattern.org). We considered *q*-value< 0.05 threshold for OncodriveFML and MutSigCV and FDR < 0.05 for MutPanning. Genes were considered as significantly mutated if they were identified by more than two tools except for *PBRM1* gene, which was detected by MutPanning and has high number of SV breakpoints.

### Detection of mutations in the promoter of *TERT* and estimation of telomere length

We identified *TERT* promoter mutations using a pileup approach to determine the number of bases that were variant from the reference. The *TERT* promoter positions assessed were c.−57 T>G (chr5:1295161), c.−124 G>A (chr5:1295228), c.−138 G>A (chr5:1295242), c.−139 G>A (chr5:1295243) and c.−146 G>A (chr5:1295250) which were previously reported as frequently mutated in mesothelioma [29, 30] and/or melanoma [31, 32]. A mutation was considered present if the mutant allele frequency was > 10%. We estimated the telomere length for tumour samples relative to the matched normal sample by counting telomere motifs in the whole genome data using qmotif [33].

### Mutational signatures

Mutational signature analysis was performed using the COSMIC [34] V3 signatures and the R package YAPSA [35] (version 1.14.0). Single base substitutions and INDEL signatures were assigned to the Alexandrov [36] SNV PCAWG with artefacts and INDEL PCAWG signatures respectively, supplied with YAPSA and downloaded August 16th, 2018. A cut-off of 10% was used to determine signature exposure.

### Estimation of homologous recombination repair deficiency

To determine if samples were HR deficient two tools were used, HRDetect [37] and HRD-score [38]. HRDetect was run with SNV mutational signatures identified with deconstructSigs [39] and SV mutational signatures from YAPSA [35]. Samples were considered HR deficient if they were predicted by both tools (HRDetect > 0.7 and HRD-sum > 42).

### TCGA cohort data analysis

TCGA MPM exome and transcriptome data for 74 previously described [4] cases were downloaded in BAM format, converted to Fastq format, and sequence reads were analysed using the same pipeline as for the Creaney et al. samples. Single nucleotide substitution variants and indels were detected using the same pipeline as the whole genome analysis. Structural variants were not called in the exome data from TCGA. Copy number was determined using sequenza [40].

Two of the cases that underwent WGS in our cohort (the Creaney et al. cohort) were previously exome sequenced and reported by TCGA (MESO1701 corresponds to TGCA-UD-AABZ and MESO2041 corresponds to TCGA-UD-AAC4) [4]. We used qsignature [41] to confirm the WGS and exome sequencing data was from the same individuals. When presenting genome data from TCGA, we excluded the two cases from TCGA and only reported the WGS data. We performed a comparison of the mutations detected by WGS and TCGA for these two cases (Additional file 2: Fig. S1).

### HLA typing and neoantigen prediction

Optitype (v1.3.1) [42] was used to compute class I HLA genotypes for paired tumour-control whole genome datasets using default parameters. Somatic variants were annotated for wildtype and mutant peptide sequences with Ensembl variant effect predictor (v86) (VEP) [43]. High confidence somatic coding variants were used to predict neoantigens using pVAC-Seq (v4.0.10) [44] and NetMHCpan [45]. Epitopes with binding affinity Inhibitory Concentration (IC50) ≤ 500 nM were considered to be potential neoantigens that bind to HLA alleles. Expressed neoantigens (IC50 ≤ 500 nM) were identified using qbasepileup [46] run in SNP mode to count the reference and mutant bases at each mutation position in the RNA-seq BAM files. Duplicate and poorly mapped reads were excluded and a mutation was considered to be expressed if there was a minimum of 10 reads with evidence of the mutation.

Gene fusion events from RNA-seq data were predicted using STAR-Fusion [47] (v1.10.0) with default settings. Predicted gene fusion events were filtered to include only those with evidence of the SV in WGS data; the remaining events were annotated using AGFusion [48] with commands ‘--middlestar’ to indicate the fusion position in the fusion peptide sequence and ‘--noncanonical’ to annotate the fusion with information from all possible transcripts. Annotated gene fusion events were used as input for pVACfuse, which is a component of pVACtools [44] to predict neoantigens using NetMHCpan (v4.0) algorithm [45] with MHC-class-I alleles.

### Whole transcriptome sequencing and analysis

Sequence libraries were generated from 1 μg intact RNA using the TruSeq stranded mRNA kit (Illumina, San Diego, CA, USA) from tumour samples. Transcriptome paired-end sequencing reads of 100 bp were generated using a Hiseq 2500 instrument (Illumina, San Diego, CA, USA) to a targeted depth of 100 million reads per sample. Adapter sequences were removed using Cutadapt [16] (v1.11) and aligned using STAR [49] (v2.5.2a) to the GRCh37 assembly and Ensembl v75 gene model. Quality control metrics were computed using RNA-SeQC (version 1.1.8), and gene expression as TPM was estimated using RSEM [50] (v1.2.30). Further analysis was carried out using TPM gene expression values to facilitate cross sample comparisons, deconvolution of immune cells and estimation of cytolytic score.

### Analysis of the tumour microenvironment (TME)

The proportion of different immune cells in the tumour micro-environment was estimated from RNA-seq data using CIBERSORT [51]. The TPM counts for the Creaney et al. and TCGA [4] datasets were calculated independently and used for deconvolution of the tumour microenvironment on a per sample basis. The CIBESORT algorithm was run with default settings, excluding quantile normalization, for 100 permutations with the published LM22 reference signature matrix to estimate the abundance of 22 immune cells types. The proportion of estimated immune cells for the Creaney et al. and TCGA [4] datasets was visualized together. Cytolytic activity was calculated for each sample as geometric mean of *GZMA* and *PRF1* expression (using TPM, 0.01 offset) as previously described [52].

### Statistical and survival analysis

All statistical analyses were performed in the R (v3.6.0) environment. A *p* value of less than 0.05 estimated using the Wilcoxon test was considered significant. Survival analysis was performed using patient overall survival and a log rank test. To determine an association with survival and the presence of whole genome duplication (WGD) a log rank Mantel-Cox test was performed. Survival analysis for neoantigen load and cytolytic activity was performed by separating samples into quartiles and comparing low and high quartiles. Survival analysis for the expression of specific genes (*TGFB1*, *CCL2*, *MMP2* and *MMP14*) was performed by stratifying samples into lower, middle and upper tertiles of log-transformed TPM+1 gene expression values. Kaplan-Meier survival curves and calculation of *p* value from a log-rank test were performed using the Survival R package (v3.3-1).

## Results

### Whole genome analysis of malignant pleural mesothelioma

**Fig. 1:**
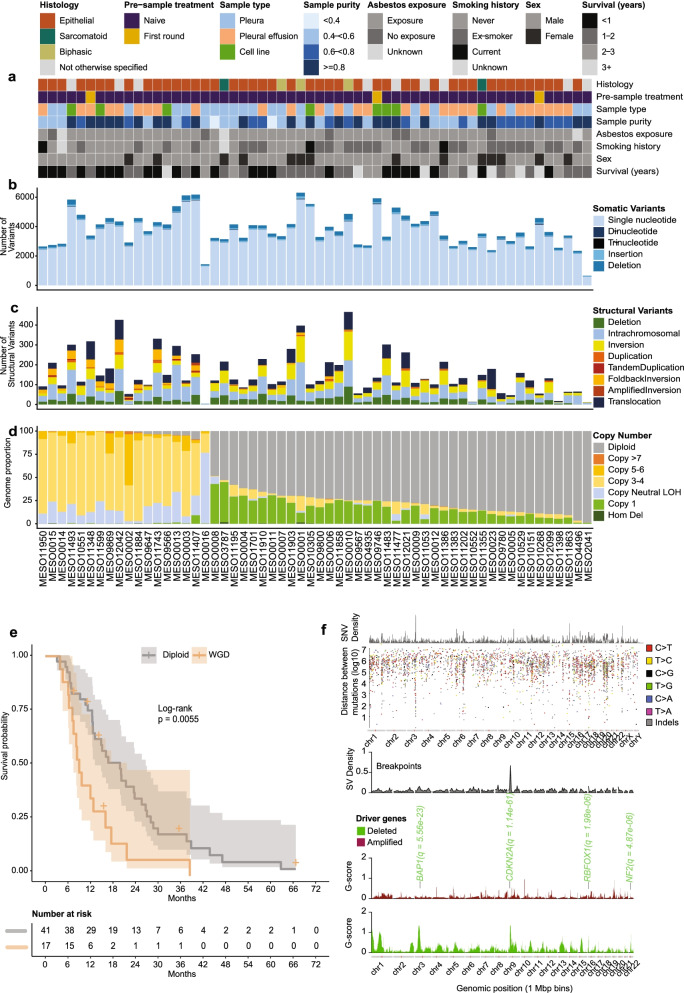
Whole genome somatic mutation burden for the Creaney et al cohort. **A** Clinical features of the 58 mesothelioma samples that underwent WGS. **B** Somatic variant load per megabase from single (SNV), dinucleotide (DNP) and trinucleotide (TNP) substitutions and short insertion and deletion (indels) variants. **C** Counts of structural rearrangements identified in each sample categorized by SV type. **D** Proportion of each tumour genome with copy number alteration (CNA). Evidence of whole genome duplication for 17 samples with > 90% of genome with CNA and > 70% of genome copy number 3-6 and a ploidy of > 2.7. **E** Kaplan-Meier curve showing overall survival was reduced in patients with evidence of whole genome duplication. **F** Density of mutations within the genomes of the 58 mesothelioma samples. Each plot is ordered by chromosome (*x*-axis). Plots show from top to bottom: genomic density of SNV and indel mutations; genomic density of SV breakpoints; frequency of amplifications (red) and deletions (green) within samples

Whole genome sequencing of 58 MPM and matched germline samples (Additional file 1: Table S1) was generated to an average read depth of 66.7 (49.7–83.6) for tumour samples and 33.7 (23.5–43.7) for matched germline samples (Additional file 3: Table S2). Data from the 58 MPM samples is referred to as the ‘Creaney et al.’ data throughout the manuscript. MPM samples included pleural tissues, pleural effusions and cell lines derived from short-term effusion cultures. The patients were predominantly male (76%, 44/58), with an average age of diagnosis of 68 years (range 43 to 95 years). Fifty percent of patients were current or previous smokers with an average of 17 ± 20 pack year smoking history, and 85% reported previous asbestos exposure (Fig. 1A and Additional file 1: Table S1). A median of 3286 (range 594 to 5855) somatic SNV and 274 (range 55 to 484) indels were detected per sample (Additional file 3: Table S2 and Additional file 4: Table S3). The average mutation burden was 1.36 mutations per megabase (median 1.31, range 0.24 to 2.24) (Fig. 1B and Additional file 3: Table S2), which is consistent with previous studies reporting a burden of < 2 mutations per megabase [3, 4]. We detected a total of 9025 somatic structural variants (SVs) (Additional file 5: Table S4) including insertions and deletions, duplications, inversions and translocations, with a median of 125 events per sample (range 5 to 466) (Fig. 1C). The majority (59%) of breakpoints occurred in intergenic regions, with 38% occurring within introns and < 3% in exons of genes. There were 159 predicted in frame gene fusion events; however, none were recurrent between samples. There were 8 genes identified in fusion events in multiple samples; however, each event was unique with no common gene partners or exon usage patterns.

Whole genome duplication (WGD) was a common event, occurring in 29% (17 of 58) of cases (Fig. 1D). WGD was significantly associated with a decrease in overall survival (*p*-value < 0.01, Log rank Mantel-Cox) (Fig. 1E). In one pleural tissue sample, loss of heterozygosity (LOH) affected almost 80% of the genome (Fig. 1D). Genomic near-haploidization, that is a genome wide LOH, has been previously described for five MPM tumours [4] which were all *BAP1*, *PBRM1* and *SETD2* wildtype, with all of the cases containing inactivating point mutations in, or homozygous deletion of, *SETDB1*. In agreement with this, the sample with genome wide LOH in our cohort was also *BAP1*, *PBRM1* and *SETD2* wildtype with a *SETDB1* inactivating mutation (frameshift deletion, chr1:150923937 AGAAG>-). The patient was male, diagnosed at 50 years of age, had no recorded exposure to asbestos and had never smoked.

Frequent SNV and indel events occurred around *BAP1* (chr3). Significant focal copy number changes as identified by GISTIC analysis (*q*-value of < 0.001) occurred on chromosomes 3, 9, 16 and 22 in regions that contain tumour suppressor genes, *BAP1* (chr3), *CDKN2A* (chr9) and *NF2* (chr22) (Fig. 1F). *RBFOX1* at chromosome 16 also contained a high number of breakpoints with a total of 67 SV events in 31 patients (Additional file 5: Table S4).

### Mutational signatures from WGS

**Fig. 2:**
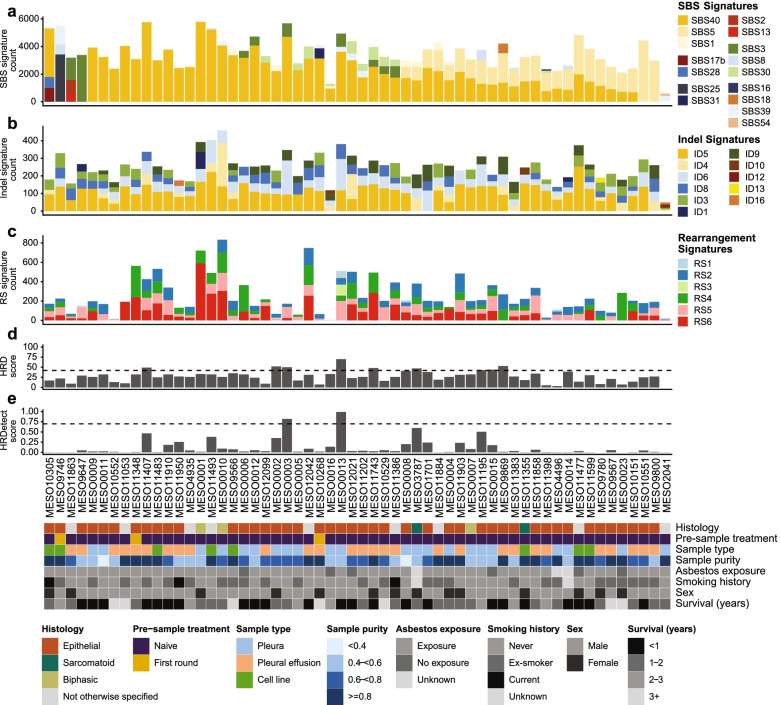
Mutational signatures and homologous recombination deficiency scores within MPM. **A** The number of single nucleotide mutations contributing to each single base substitution (SBS) somatic mutational signature within each patient that was identified using COSMIC v3 signatures. The major common categories are grouped together using similar colours. **B** The number of indel mutations contributing to each indel signature within each patient. Somatic indel mutational signatures estimated from indels with a size of 1 to 50pb and compared to COSMIC v3 signatures using cosine similarity. **C** The number of SV mutations contributing to each rearrangement signature within each patient. **D** The HRD-sum score for each patient. The dashed line represents the threshold for HR deficiency. **E** The HRDetect score for each patient. The dashed line represents the threshold for HR deficiency. Sample and patient features are shown below the plots

A mutation signature associated with asbestos exposure has not been identified, therefore we hypothesized that the increased unbiased coverage of the genome afforded by WGS would provide more power to detect signatures. When assigning to the known signatures from COSMIC database v3 [34, 36] using the 203,416 somatic SNVs and 15,864 somatic indels from the 58 samples that underwent WGS, we identified 16 single base substitution signatures (SBS) (Fig. 2A and Additional file 2: Fig. S2) and 11 indel signatures (ID) (Fig. 2B and Additional file 2: Fig. S3). In addition a total of 6 SV signatures were identified (Fig. 2C).

The majority of samples (54 of 58) contained either SBS5 or SBS40 signature at a proportion of > 50%. Both these signatures have been described as flat signatures that are similar to one another and present in multiple cancer types [36], which means their relative assignment may be uncertain. SBS40 and SBS5 have been shown to correlate with age of diagnosis in some cancer types [36]. Although there was a trend (*p* 0.035), we did not see a strong correlation of SBS40 with age in our cohort (Additional file 2: Fig. S4A). The indel signature ID5, which was present in most samples (38 of 58) at a proportion of > 40%, showed a stronger association with age (Additional file 2: Fig. S4B). Previously, it has been proposed that the mutational processes that underlie the age-correlated signatures SBS40 and ID5 are similar [36]; however, we did not see a correlation in the abundance of these signatures in the mesothelioma cohort. Additionally, the proportion of SBS40 or SBS5 signatures within patients were not associated with asbestos exposure, smoking history or age of diagnosis.

The four samples that did not contain SBS40/5 signatures had signatures that were unique or infrequent within the cohort (Fig. 2A). The first sample (MESO09746) was comprised of SBS8, SBS25 and SBS39. The SBS39 signature was present in 6 other samples, and currently, there is no known aetiology for this signature. SBS8 has been implicated in homologous recombination deficiency (HRD); however, we could not find any mutations in the *BRCA* genes of MESO09746, and this sample had a low HRD-sum score (Fig. 2D) and HRDetect score (Fig. 2E) suggesting the sample was not HR deficient. SBS25 has been associated with chemotherapy treatment [36], and this sample had undergone chemotherapy treatment. Similarly, MESO10268 was also pretreated and contained SBS31 also associated with platinum compound chemotherapy [36]. The second sample that did not contain SBS40/5 (MESO2041) was comprised of SBS39 and SBS54, the former has no known aetiology, while SBS54 has been described as a sequence artefact possibly due to germline variant contamination. This sample had the lowest number of mutations within the cohort (594 SNVs) which has impacted the ability to confidently resolve signatures (Additional file 2: Fig. S2A). The third sample (MESO11863) contained a germline *BAP1* mutation, and was comprised of the APOBEC linked signatures SBS2 and SBS13, as well as SBS3. In the fourth sample lacking SBS40/5 (MESO9647), the only signature detected was SBS3.

The SBS3 HR deficient signature has previously been associated with HRD and germline and somatic *BRCA1* and *BRCA2* mutations and *BRCA1* promoter methylation in breast [53], pancreatic [54] and ovarian [23] cancers. SBS3 was present in 9 samples, with low levels (< 30%) in 7 samples (Fig. 2A). Two of these samples (MESO0013 and MESO0003) also contained a high ID6 signature and high HRD-sum and HRDetect scores suggesting these samples are HR deficient. A somatic frame-shift mutation in *BRCA1* (NM_007294.4:c.1504_1508del (p.Leu502fs)) with LOH of the other allele accounts for the signature in MESO0013, while MESO0003 contains a somatic SV break within the *BRCA2* gene which potentially results in a disruption of the gene. These findings suggest a low prevalence of HR deficiency in a subset of MPM. Another DNA damage signature present at low prevalence (< 30%) in 10 samples was SBS30 which has been associated with base excision repair deficiency due to inactivating mutations in *NTHL1* [55]. However, we did not detect *NTHL1* mutations in our cohort.

### Somatic driver gene analysis and frequently disrupted genes

**Fig. 3:**
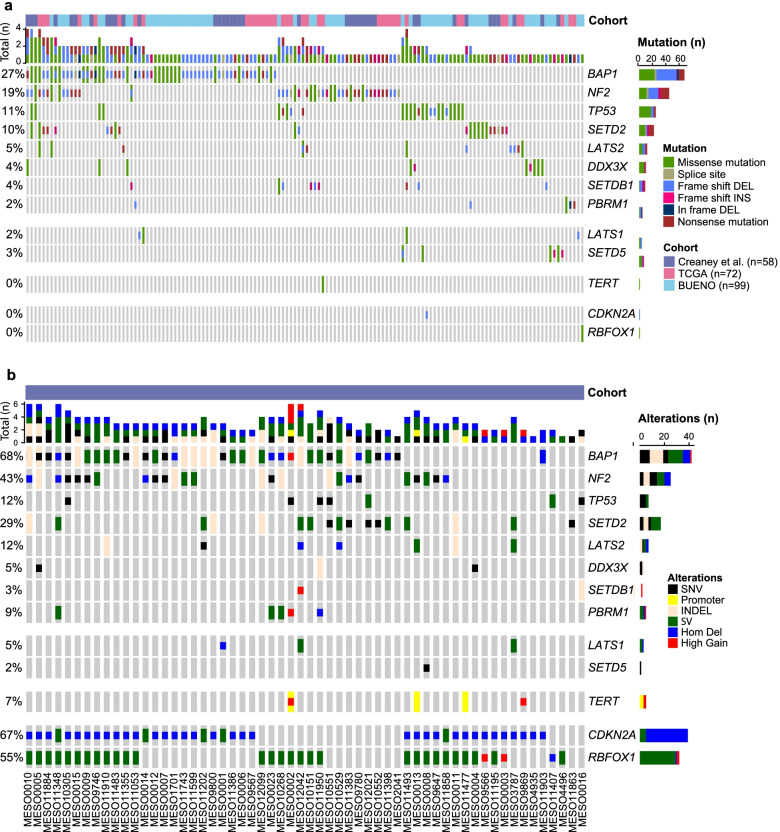
Overview of genomic alterations in driver genes of MPM. Genes are shown if they were identified as significantly mutated, have recurrent promoter mutations, were present in GISTIC analysis or contained a high number of breakpoints. **A** Somatic SNV and indel mutations across three cohorts. The colour bar at the top indicates the sample cohort: Creaney et al. (purple), TCGA (pink) and Bueno et al. (light blue). The histogram shows the number of mutated driver genes in each sample. The oncoplot shows the mutations in each gene. The number of samples with mutations in each gene is shown in the bar chart on the right. The type of SNV or indel mutation is shown using colour codes. **B** Analysis is restricted to the samples in the Creaney et al. cohort which underwent WGS. The histogram shows the number of mutated driver genes in each sample. The oncoplot shows the SNV and indel mutations, breakpoints and copy number alterations in each gene. The order of each gene is the same as in panel A, and the sample labels are along the bottom. The mutation type is shown using colour codes

Driver oncogenes for MPM have not been previously identified. In order to generate increased power for identifying such genes, we combined the protein coding substitution and indel variants in our cohort with published studies from TCGA [4] and Bueno et al. [3] and used three approaches to identify significantly mutated genes (SMG). The driver gene meta-analysis of 229 patients identified 7 driver genes (*BAP1, NF2, TP53, SETD2, LATS2, DDX3X* and *SETDB1*) which were significant in two SMG approaches (*q*-value of < 0.05) (Fig. 3A and Additional file 6: Table S5), all of which have previously been reported as MPM tumour drivers [3, 4]. Another gene, *PBRM1*, which is known to frequently undergo copy number loss in MPM [56] was found to be significantly mutated by only one approach (Additional file 6: Table S5). Interestingly, the meta-analysis highlighted low frequency mutations in *LATS1* and *SETD5* genes, which although not significant are family members of the known MPM driver genes (Fig. 3A). A significantly mutated or candidate driver gene was detected in 61.2% (140/229) of the samples (Fig. 3A).

In addition to this, we analysed the Creaney et al. WGS dataset in isolation to incorporate the WGS-derived gene breakage (SV), copy number alterations (CNA) and gene promoter mutations. The chromosome breakpoints increased the mutational frequency of *BAP1*, *NF2*, *TP53*, *SETD2*, *LATS2* and *LATS1* (Fig. 3B) and highlighted other recurrently affected genes including *CDKN2A* (which was also significant by GISTIC (Fig. 1F)) and *RBFOX1*. We also found mutations in the promoter of the *TERT* gene in 3 of 58 samples (5.2%) (Fig. 3B) confirming previous reports of frequent mutations in the *TERT* promoter [29, 30, 57, 58]. The presence of *TERT* promoter mutations was not associated with a high tumour mutation burden, a low SV number or relative tumour telomere length (Additional file 3: Table S2). In agreement with previous reports [30], we found that *TERT* mutations were mutually exclusive to *BAP1* mutation, as the 3 samples with *TERT* promoter mutation and an additional sample with *TERT* amplification did not contain loss of function *BAP1* mutations (Fig. 3B).

The RNA-seq data was used to assess the impact of mutation on expression of these genes. There was a significant difference in expression of *BAP1* between *BAP1* mutated and wild type samples in the WGS pleura (*p* 0.042) (Additional file 2: Fig. S5), but not in the samples from TCGA (Additional file 2: Fig. S6). One sample (MESO1147) was wild type for *BAP1* mutation but contained a low expression of *BAP1*. There was also a significant association with mutation and low expression for *NF2* in samples from cell lines (*p* 0.042), pleura (*p* 0.00017) and effusions (*p* 0.0047). Although only significant in effusion samples (*p* 0.005) and TCGA data (*p* 0.038), the majority of samples of both cohorts with a loss of function mutation in *CDKN2A* also contained lower expression compared to wild type samples.

### Neoantigens in MPM tumours

**Fig. 4:**
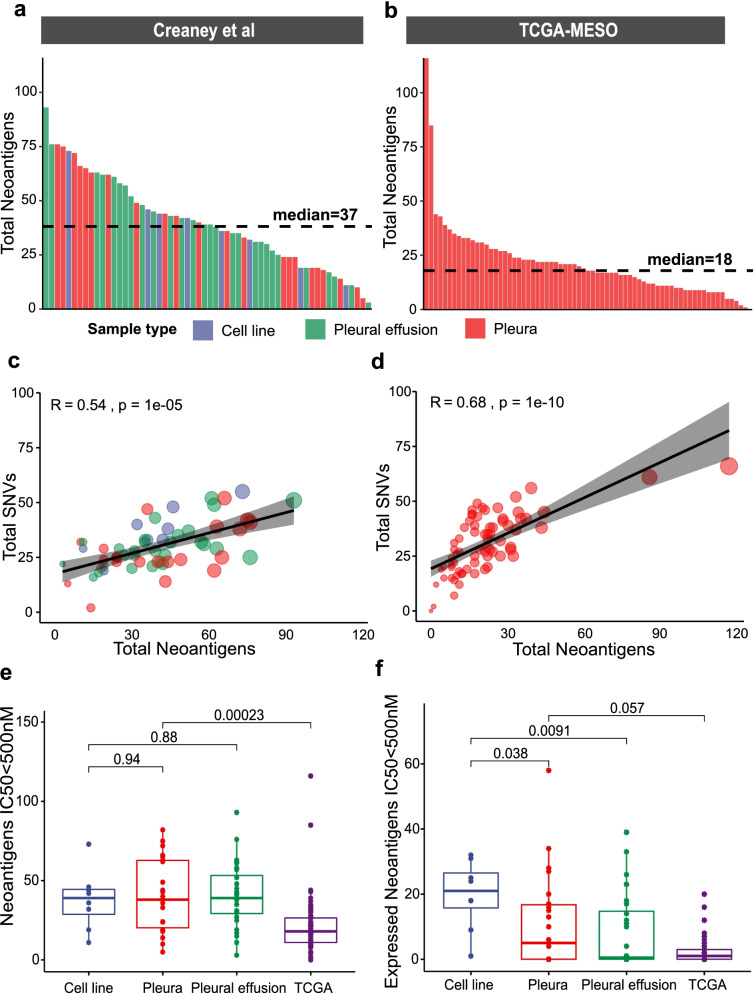
Neoantigen load derived from SNV and indel mutations in MPM. Predicted total neoantigen load derived from SNVs and indels with IC50 ≤ 500 nM in the **A** Creaney et al. cell line, pleura and pleural effusion samples and **B** TCGA pleura samples. Pearson correlation plots between short nucleotide variants (SNVs) on *x*-axis and neoantigen load on *y*-axis for **C** Creaney et al. and **D** TCGA dataset. **E** Boxplots for predicted total neoantigen load (IC50 ≤ 500 nM) (y-axis) in Creaney et al. cell line, pleura and pleural effusion samples and TCGA pleura samples (x-axis). **F** Boxplots for expressed neoantigen load (IC50 ≤ 500 nM) in Creaney et al. samples and TCGA pleural samples. *p*-values shown are from Wilcox test

Somatic mutations may create tumour specific neoantigens that could be important in immune recognition. We used the SNV and indel somatic mutations to predict and found a median of 37 neoantigens per sample (range 3–93) in the Creaney et al. WGS data presented in this manuscript (Fig. 4A) and 18 neoantigens per sample (range 1–116) in data from TCGA (Fig. 4A). The number of predicted neoantigens correlated with number of SNV in the Creaney et al. (Fig. 4C) and TCGA data (Fig. 4D). The Creaney et al. cohort was comprised of samples derived from the pleura, effusions or short-term cell cultures, but the neoantigen load was not associated with sample type (Fig. 4E). The neoantigen load in the pleural tissue samples from the Creaney et al. cohort was significantly higher than TCGA (*p* 0.00023, Fig. 4E). This difference may be associated with differences in tumour purity between the cohorts or different sequencing approaches possibly due to WGS having the potential to produce more even read depth across the genome and may include coding regions not targeted by the exome sequencing. The RNA-seq was used to determine which of the somatic mutations that were predicted to form neoantigens were expressed. The short-term cell line samples contained significantly more expressed neoantigens (*p* < 0.05, Fig. 4F) possibly due to their high tumour content. Very few neoantigens were predicted from somatic indels (Additional file 2: Fig. S7A and Fig. S7B). In contrast with other cancers [59], there was no association between indel neoantigen load and overall survival in the Creaney et al. or TCGA data (Additional file 2: Fig. S7C and Fig. S7D).

Gene fusion events represent another potential source of neoantigens. We identified a total of 159 candidate gene fusion events in the 58 samples (Additional file 5: Table S4); 53 were caused by inter-chromosomal events (Additional file 2: Fig. S8A) and 106 by intra-chromosomal events (Additional file 2: Fig. S8B); of these, 13 were detected in the RNA-seq (Additional file 2: Fig. S8C). Only 6 of these fusion events were predicted to result in the formation of neoantigens (Additional file 2: Fig. S8D).

### Immune cells within the MPM microenvironment

**Fig. 5:**
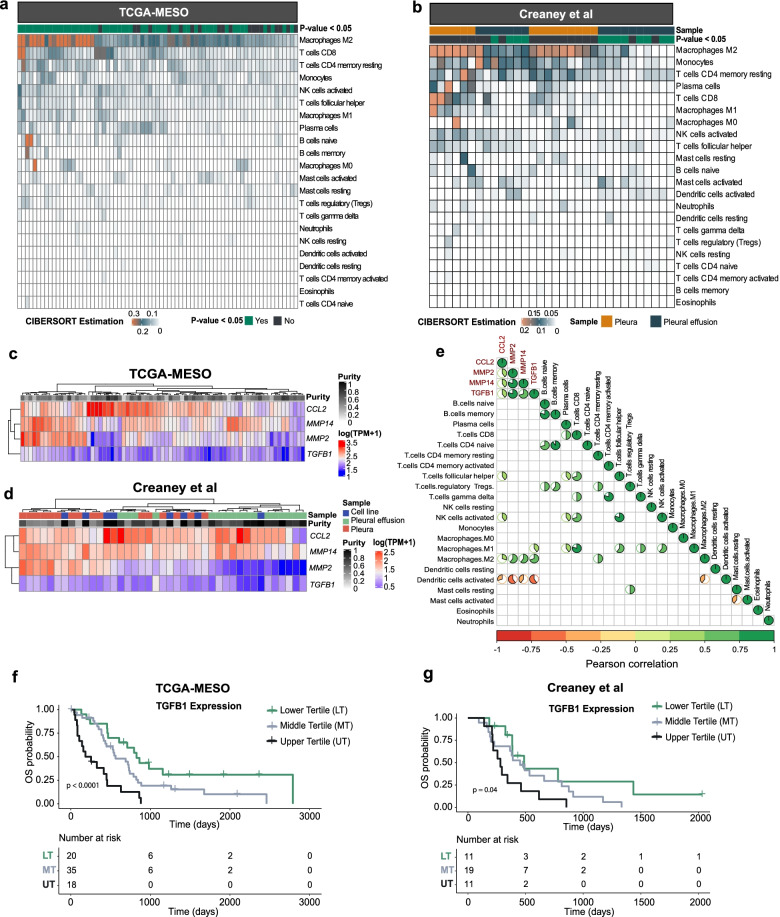
The tumour micro-environment of MPM. Deconvolution of immune cells in the tumour micro-environment (TME) for **A** pleura samples of TCGA and **B** pleura and effusion sample of Creaney et al. Samples are on *x*-axis and the estimated proportion of immune cells is on the *y*-axis. Sample information and CIBERSORT estimated *p*-value for enrichment of immune cells per sample are shown in the tiles above the plot. RNA-seq data for **C** TCGA and **D** Creaney et al. showing the log transformed TPM +1 gene expression values for *CCL2*, *MMP2*, *MMP14* and *TGFB1*. **E** Correlation of log transformed TPM+1 gene expression values of *CCL2*, *MMP2*, *MMP14* and *TGFB1* and the proportion of immune cells measured with CIBERSORT. The correlation was estimated using Pearson Correlation as indicated in the scale bar. Values are shown for 66 samples (53 TCGA and 13 Creaney et al.) which have significant immune cell proportions estimated by CIBERSORT (*p*-value < 0.05). Comparisons that are significantly correlated (*p*-value < 0.001) with a positive correlation are displayed in green pie charts and negative correlation are displayed in red pie charts. Blank panels indicate a non-significant correlation (*p*-value > = 0.001). **F** Kaplan-Meier plot of TCGA samples with *TGFB1* expression divided by lower, middle and upper tertiles (*p* value from log rank test). **G** Kaplan-Meier plot of Creaney et al. samples with *TGFB1* expression divided by lower, middle and upper tertiles (*p* value from log rank test)

Deconvolution of the RNA-seq data to estimate immune cells using CIBERSORT revealed M2 macrophages, monocytes, CD4 and CD8 T cells in the tumour microenvironment (TME) of TCGA pleura samples (Fig. 5A) and the pleura and effusion samples of Creaney et al. (Fig. 5B). There was a lower proportion of immune cells in the pleural effusion samples of the Creaney et al. data, likely due to the samples undergoing CD45 depletion prior to genomic analysis. Generally, the most prevalent immune cell type was M2 Macrophages. However for seven samples the proportion of CD8 T cells in the TME was high (> 0.2) (Fig. 5A and B). The proportion of CD8 T cells correlated with cytolytic activity in pleura and effusion samples (Additional file 2: Fig. S9A). The cytolytic activity has been reported as a prognostic factor in a variety of cancer types [52]; however, in MPM, we did not see an association with survival (Additional file 2: Fig. S9B).

### TGFB is associated with M2 macrophages and worse survival

The secretion of chemokines and growth factors, in particular *TGFB1*, into the TME has a role in MPM development and may affect the immune cells within the TME [60]. The expression of chemokines in our data and TCGA revealed that *CCL2* was the highest expressed chemokine in all sample types (cell lines, pleural effusion and pleura) (Fig. 5C and D and Additional file 2: Fig. S10A and Fig. S10B). Similarly, matrix metalloproteases (MMPs) may promote invasion of MPM cell lines [61] and have been associated with immune desert regions in the TME [62]. We found that *MMP14* and *MMP2* were the highest expressed MMPs (Fig. 5C and D and Additional file 2: Fig. S10A and Fig. S10B).

The expression of *CCL2*, *TGFB1*, *MMP14* and *MMP2* were associated with the presence of M2 macrophages (Pearson correlation of 0.3, 0.7, 0.6 and 0.6 respectively with a *p* < 0.001) (Fig. 5E), suggesting that they may be contributing to a cold TME enriched with M2 macrophages. A low expression of *TGFB1* was associated with a better survival within the TCGA (Fig. 5F) and Creaney et al. datasets (Fig. 5G) (*p* < 0.05, log rank test), whereas low *MMP2* and *MMP14* expression was associated with better survival (*p* = 0.041 and 0.011 respectively, log rank test), but only in the TCGA data (Additional file 2: Fig. S10C).

### Expression of immune checkpoint receptors in MPM

Immune checkpoint blockade (ICB) agent anti-PD1 (Nivolumab) and anti-CTLA4 (ipilimumab) have been approved by the FDA for MPM and showed efficacy in the CheckMate 743 trial [7]. Therefore, we performed a retrospective analysis of immune checkpoint receptors to identify potential ICB targets in MPM tumours that may improve the responses to ICB therapy (Additional file 2: Fig. S11). The expression of *VSIR* which encodes the V-type immunoglobulin domain-containing suppressor of T cell activation (VISTA) was high in most samples as previously reported [4]. Similarly, high expression of *CD276* was observed in most samples. The pleura and pleural effusion samples in the Creaney et al. and TCGA datasets clustered into two groups. A small subset of samples (less than 10%) clustered into group 2 and were associated with higher cytolytic scores and high expression of immune checkpoint receptors (Additional file 2: Fig. S11) indicative of a potential T cell inflamed immune ‘hot’ phenotype, suggesting these may potentially respond to immunotherapy. Previously, loss of *BAP1* has been associated with immunogenicity in MPM [63], but here, we found no association of *BAP1* status with neoantigen load (Additional file 2: Fig. S11) or the immune hot subgroup.

## Discussion

Previous genomic studies of MPM were performed primarily using exome and transcriptome sequencing [3–5, 64], with limited whole genome studies. Here, we report a large WGS study of MPM. We confirm that MPM is driven by loss of function of tumour suppressor genes [4], and using WGS, we identify SV breakpoints disrupting key driver genes to extend the mutational spectrum of these genes. Finally, we use RNA-seq to explore the TME to create the most complete picture of MPM.

Obtaining MPM surgical tissue samples for genomic studies is challenging, as surgery does not always provide clinical benefits to all MPM patients as demonstrated by the randomized clinical Mesothelioma and Radical Surgery (MARS) trial [65]. Therefore, genomic analysis of surgical samples is generally limited to a subgroup of patients that are operable. Pleural effusions are a common early feature in most patients with epithelioid MPM and provide a ‘window’ to the underlying tumour; because the fluid is readily accessible, the fluid may capture more of the tumour heterogeneity as it contains cells shed from multiple areas of the tumour and sequential samples can be obtained. Effusion samples may contain a low proportion of tumour cells, which may inhibit their utility for WGS. We found that cell line and pleural effusion samples contain an overall mutation spectrum similar to MPM tissue samples, in keeping with previous findings [66], supporting the use of effusion samples to profile MPM. However, in our cohort, we performed tumour cell enrichment of effusion samples using low passage cell culture or CD45 depletion to improve tumour purity and detection of mutations. It should be noted that both these approaches will impact the proportion of immune cells within the TME, and future studies using single cell approaches to profile effusion samples would reveal greater insight into tumour immune composition.

Mesothelioma is a carcinogen-associated cancer linked to exposure of asbestos [1]. Several other carcinogen-associated cancers have been associated with a high tumour burden and specific mutational signatures [67] (UV-driven melanoma and smoking in lung cancer). In contrast, we found that MPM contains a modest SNV and indel mutation burden, which agrees with previous studies of MPM [3–5]. The most frequent signature observed was SBS40, which has not previously been associated with a specific aetiology and has been detected in a variety of cancer types. One could speculate that Signature SBS40 is present in MPM due to the inflammatory effects of asbestos exposure and reactive oxygen species induced DNA damage. However, this needs to be confirmed in more MPM samples or with functional studies. Several cases harboured specific signatures, which may reflect individual tumour characteristics. For example, we found one case with an APOBEC signature. Interestingly, this patient was the only case in the cohort to have a *BAP1* germline variant. However, an association with *BAP1* germline variants and the APOBEC signature has not been reported, and the lack of the APOBEC signature within samples that contained somatic *BAP1* mutations means they are likely not associated.

*BAP1* is known to be frequently disrupted in MPM [3, 4, 68], the WGS and SV breakpoints, enabled a more complete mutation picture, and we found that *BAP1* alteration occurs in over two thirds of WGS cases and was significantly associated with lower gene expression in pleura samples. In our analysis, gene expression in TCGA data was not significantly different between *BAP1* mutated and wildtype samples, possibly because exome sequence data is unable to identify all mutation events in *BAP1* such as CNA and SV. We did not assess other mechanisms of *BAP1* inactivation such as methylation that may contribute to low *BAP1* gene expression in samples without *BAP1* mutation. The prevalence of *BAP1* loss adds impetus to the search for treatments that could benefit this subgroup of patients. An in vitro study demonstrated that a *BAP1* mutant cell line responded to a combination of PARP inhibition and cisplatin [69] supporting BAP1 as target for therapy, with patient trials underway in MPM [70, 71] and other BAP1 altered tumours. These approaches either directly target BAP1 function using HDAC inhibitors and EZH2 inhibitors or target DNA damage repair mechanisms with the use of platinum chemotherapy or PARP inhibitors [6]. Results from an early stage MPM clinical trial of PARP inhibitors reported a manageable toxicity profile and partial response in 3 of 26 patients, all with BAP1 loss [70]. In breast and ovarian cancer, HR deficiency-associated mutational signatures are predictive of mutations in *BRCA1/2* and postulated to be associated with PARP inhibitor response [37]. However, in our WGS-driven analysis, we found these signatures were present at a low frequency in a small subset of mesothelioma patients, with only two samples considered as HR deficient. This suggests that PARP inhibition may only be effective in a small subset of MPM, and we await the outcomes of several PARP inhibitor clinical trials currently underway (NCT03531840 and NCT03207347).

In addition to *BAP1,* we also identified other previously reported MPM driver genes, and novel candidate low frequency MPM driver genes. *RBFOX1* was the gene most frequently disrupted by SV events, which supports previous reports [72]. Whether chromothripsis or other mechanisms causing complex rearrangements was responsible for this recurrent rearrangements is not clear, and future work to profile the chromatin landscape through methylation profiling or ATAC-seq would be useful. We also confirm previous reports of recurrent *TERT* promoter mutations, which rarely co-occur [57] or are mutually exclusive to *BAP1* mutation [30]. However, in our cohort, *TERT* promoter mutations were detected in 5.2% of samples, which is lower than previous studies reporting 15% [58], 11.6% [29] and 10.4% [30]. The difference in prevalence may be due to the MPM samples that underwent WGS in our cohort being predominantly an epithelioid histologic subtype, since *TERT* promoter mutations have been reported as enriched in sarcomatoid [58] and non-epithelioid MPM [57]. Unlike previous reports in melanoma [32], the *TERT* promoter mutations were not associated with a high tumour mutation burden, a low SV number or telomere length [32]; however, our analysis was limited as only 3 samples contained a *TERT* promoter mutation. Two novel candidate genes were detected (*LATS1* and *SETD5*) and are family members acting within the same pathway as previously reported MPM driver genes (*LATS2* and *SETDB1*) and occur in a mutually exclusive manner. MPM has similarities to clear cell renal cell carcinoma (ccRCC), as both harbour frequent loss of function mutations in *SETD2*, *SETD5* and *BAP1*, and we confirm in this study *PBRM1* [73, 74]. *PBRM1* mutations have previously been detected in two MPM cases in Bueno et al. [3] and TCGA [4]; using WGS, we also identified somatic disruptive SV and CNA events as a common mechanism to disrupt *PBRM1*. *PBRM1* and *BAP1* loss co-occurred, which may be due to both genes being located within 200 kb of each other on chromosome 3. Interestingly, *PBRM1* loss-of-function events in ccRCC have been associated with an increased response to immunotherapy checkpoint blockade [75], warranting investigation for MPM.

Checkpoint blockade has emerged as a treatment for MPM patients [7, 9]. Whether a tumour will respond to immunotherapy is impacted by both the tumour cells and the tumour immune environment. Markers to identify patients that will respond to immunotherapy will be useful in the management of MPM. We identified a small subset of patients that contained a high proportion of CD8 T cells and a strong cytolytic activity score, suggesting an immune ‘hot’ phenotype that may be more amenable to immunotherapy. Loss of *BAP1* has been described as a candidate predictive marker of immunotherapy response in MPM [76] and in peritoneal mesothelioma *BAP1* loss has been linked to an inflammatory tumour microenvironment with increased T cell infiltrate [63]. Our findings did not support a link with *BAP1* loss and the immune ‘hot’ phenotype in MPM. Compared to peritoneal mesothelioma, MPM harbour more frequent loss of *CDKN2A*, and loss of *CDKN2A* has been linked to resistance to immunotherapy in non-small cell lung cancer [77]. Therefore, the *CDKN2A* loss may contribute to the lack of association with the immune ‘hot’ phenotype in MPM. The most abundant immune cell type in MPM samples was M2 macrophages, suggesting an immunosuppressive phenotype [78]. *CCL2* is involved in the polarization of monocytes into M2 macrophages in the TME of a variety of cancers [79] including MPM [80], in agreement with this we detected high expression of *CCL2*. Similarly, we confirmed high *TGFB1* expression, which is also involved in M2-like macrophage polarization [81], and an association with a poorer outcome [82]. The clinical role of these observations is the subject of ongoing studies.

Chemokine-related treatment avenues have been suggested as a potential opportunity of therapy and given their high expression may be relevant to MPM. Potential treatment approaches include targeting the CCL2-CCR2 axis [83] or blocking TGF-β [84]. Blocking TGFβ has previously shown a potential survival benefit in MPM [85]. Tumour vaccines targeting neoantigens is another emerging therapy [86]. However, as the mutation profile of MPMs has a high interpatient heterogeneity, neoantigen vaccine approaches may need to be personalized.

## Conclusions

**Fig. 6:**
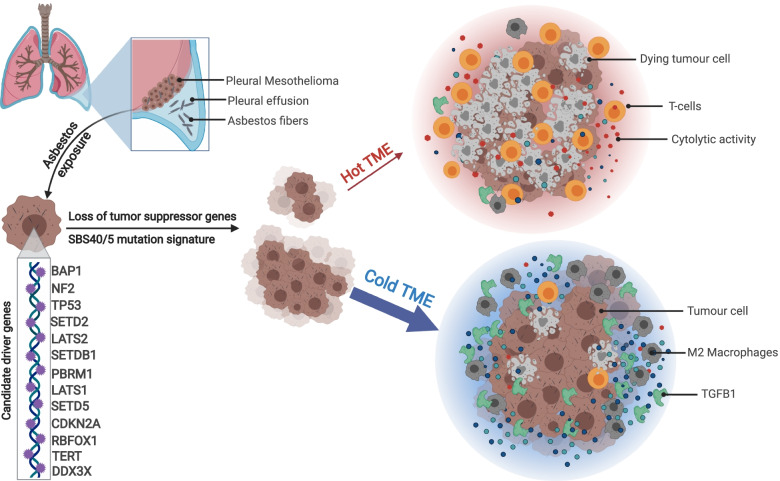
An overview of key mutation processes and the tumour microenvironment in MPM. Inhaled asbestos fibres located at parietal pleura of lung. Asbestos fibres may trigger cell damage and contribute to initiation of mesothelioma cells. Whole genome sequencing of mesothelioma samples revealed 13 candidate driver genes and mutations were enriched with SBS40/5 mutation signature. Whole transcriptome sequencing identified ‘hot’ a TME marked with the presence of T cells and cytolytic activity in a subset of samples. The majority of MPM favour the growth of the tumour cells by promoting a "cold" TME comprised of M2 Macrophages and *TGFB1* expression. Figure created with BioRender.com

In summary, we extend the mutational analysis of MPM using WGS. By combining our data with existing datasets [3, 4] we confirmed previously described driver genes and identified candidate new driver genes. Integration of the transcriptome revealed the complexity of predicting patient treatment response and highlighted the importance of the tumour environment. We show that MPM is driven by mutations in tumour suppressor genes and frequently contains the SBS40 mutational signature. The tumours contain a high M2 macrophage infiltrate within the pleura and effusion TME, which is linked to high *TGFB1* and *CCL2* expression. A small subset of samples displayed an immune ‘hot’ phenotype with CD8 T cells and high cytolytic activity (Fig. 6). We propose that, depending on the immune microenvironment, patients may respond to immune checkpoint and/or TGFB blockade. Further genomic analysis of MPM immunotherapy clinical trial samples will be key to better understanding the potential molecular biomarkers and ultimately improving outcomes for patients.

## Supplementary Information

**Additional file 1: Table S1.** Clinical characteristics of the MPM samples that underwent whole genome sequencing

**Additional file 2: Figure S1.** A comparison of somatic mutations detected by WGS and exome sequencing; **Figure S2.** Single base substitution somatic mutational signature identified in MPM; **Figure S3.** Somatic short indel mutational signatures identified in MPM; **Figure S4.** Spearman correlation of age of diagnosis with mutational signatures; **Figure S5.** Gene expression of MPM genes in tumours with and without mutation in the Creaney at al. cohort; **Figure S6.** Gene expression of MPM genes in tumours with and without mutation in TCGA cohort; **Figure S7.** Indel neoantigen load in MPM; **Figure S8.** Gene fusion events and neoantigen prediction in MPM; **Figure S9.** Cytolytic activity and survival in MPM; **Figure S10.** Chemokine, cytokine, interleukin and matrix metalloproteases expression in MPM; **Figure S11.** Expression of immune checkpoint receptors in MPM.

**Additional file 3: Table S2.** Summary of read depth and the number of somatic mutations identified in MPM samples from whole genome sequencing.

**Additional file 4: Table S3.** Somatic coding mutations detected in the 58 MPM.

**Additional file 5: Table S4.** Somatic structural rearrangements detected in the 58 MPM samples.

**Additional file 6: Table S5.** Significantly mutated gene analysis in MPM.

## Data Availability

All sequence data have been deposited in the European Genome-phenome Archive (EGA) repository under the study EGAS00001005196, https://ega-archive.org/studies/EGAS00001005196 [87] with datasets for the RNA sequence data (EGAD00001007874) and whole genome sequence data (EGAD00001008341 and EGAD00001008447). The following tools are available in GitHub: qcoverage https://github.com/AdamaJava/adamajava/tree/master/qcoverage [19], qsignature https://github.com/AdamaJava/adamajava/tree/master/qsignature [41] and qbasepileup https://github.com/AdamaJava/adamajava/tree/master/qbasepileup [46].
