# Supplementary material for: Comprehensive genomic and tumour immune profiling reveals potential therapeutic targets in malignant pleural mesothelioma
Source: Genome Med. 2022 May 30;14:58. doi: 10.1186/s13073-022-01060-8 (PMC9150319; doi:10.1186/s13073-022-01060-8)
Supplement: Supplementary file 2 — Additional file 2: Figure S1. A comparison of somatic mutations detected by WGS and exome sequencing; Figure S2. Single base substitution somatic mutational signature identified in MPM; Figure S3. Somatic short indel mutational signatures identified in MPM; Figure S4. Spearman correlation of age of diagnosis with mutational signatures; Figure S5. Gene expression of MPM genes in tumours with and without mutation in the Creaney at al. cohort; Figure S6. Gene expression of MPM genes in tumours with and without mutation in TCGA cohort; Figure S7. Indel neoantigen load in MPM; Figure S8. Gene fusion events and neoantigen prediction in MPM; Figure S9. Cytolytic activity and survival in MPM; Figure S10. Chemokine, cytokine, interleukin and matrix metalloproteases expression in MPM; Figure S11. Expression of immune checkpoint receptors in MPM. [file 13073_2022_1060_MOESM2_ESM.docx]

**Comprehensive genomic and tumour immune profiling reveals potential therapeutic targets in malignant pleural mesothelioma**

Jenette Creaney** ^3,4,5^, Ann-Marie Patch** ^1,2^, Venkateswar Addala** ^1,2^, et al.

This document contains Figures S1-11.

**Fig. S1.** A comparison of somatic mutations detected by WGS and exome sequencing

**Fig. S2.** Single base substitution somatic mutational signature identified in MPM

**Fig. S3.** Somatic short indel mutational signatures identified in MPM

**Fig. S4.** Spearman correlation of age of diagnosis with mutational signatures

**Fig. S5.** Gene expression of MPM genes in tumours with and without mutation in the Creaney at al. cohort

**Fig. S6.** Gene expression of MPM genes in tumours with and without mutation in TCGA cohort

**Fig. S7.** Indel neoantigen load in MPM

**Fig. S8.** Gene fusion events and neoantigen prediction in MPM

**Fig. S9.** Cytolytic activity and survival in MPM

**Fig. S10.** Chemokine, cytokine, interleukin and matrix metalloproteases expression in MPM

**Fig. S11.** Expression of immune checkpoint receptors in MPM

**
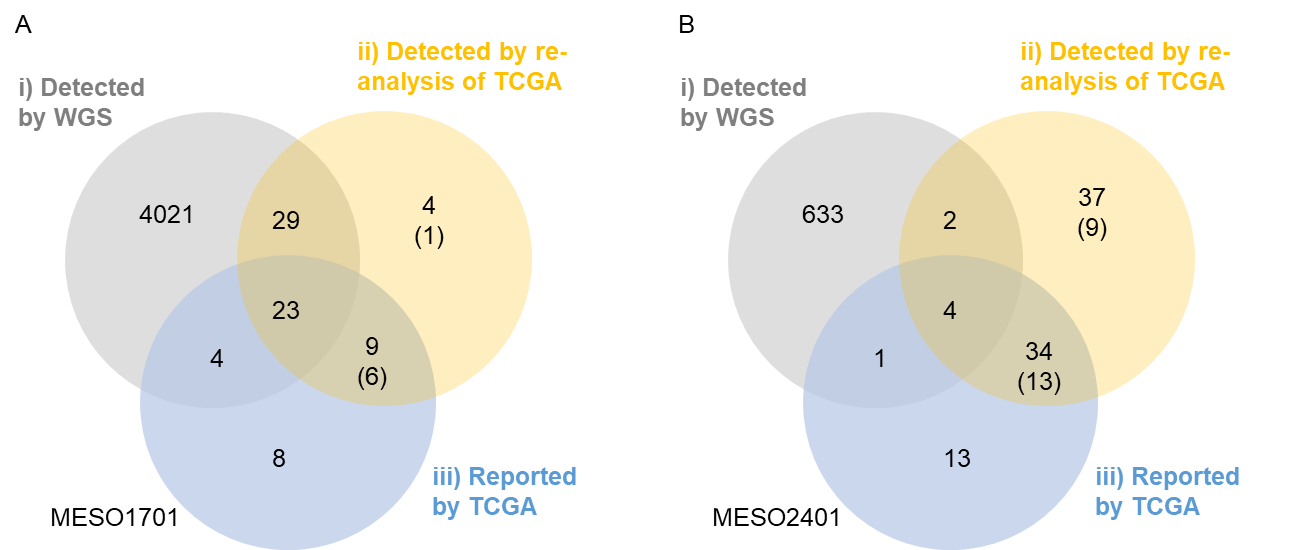
**

**Fig. S1. A comparison of somatic mutations detected by WGS and exome sequencing.** Two of the mesothelioma cases profiled by WGS in this study were previously exome sequenced by TCGA: MESO1701 matches TGCA-UD-AABZ; and MESO2041 matches TCGA-UD-AAC4. We performed a comparison of somatic mutations detected in the WGS and TCGA exome data by comparing i) Somatic mutations detected by WGS; ii) Somatic mutations from our analysis of TCGA data; and iii) Somatic mutations reported by TCGA (accessed from the NCI GD data portal in March 2022). A) Venn diagram showing the overlap for MESO1701 (TGCA-UD-AABZ), and B) Venn diagram showing the overlap for MESO2041 (TCGA-UD-AAC4). Somatic mutations that were called in the re-analysis of TCGA data and not called by WGS underwent a pileup approach, whereby we mapped the variant position to the WGS data and counted the reads with and without the variant. This enabled identification of variants present at low level in the WGS data but not called in the WGS, the number of these variants is shown in the brackets. The pileup approach was used determine likely germline variants (those present in the non-tumour (normal) WGS BAM files). The likely germline variants and mutations located on the mitochondrial chromosomes or on contigs were excluded from the comparison. We suspect the low overlap of mutations in B) between the exome analyses and WGS is associated with different parts of the tumour tissue being sequencing by TCGA and WGS as well as low sequence quality in TCGA exome data, possibly driven by poor DNA quality.

**
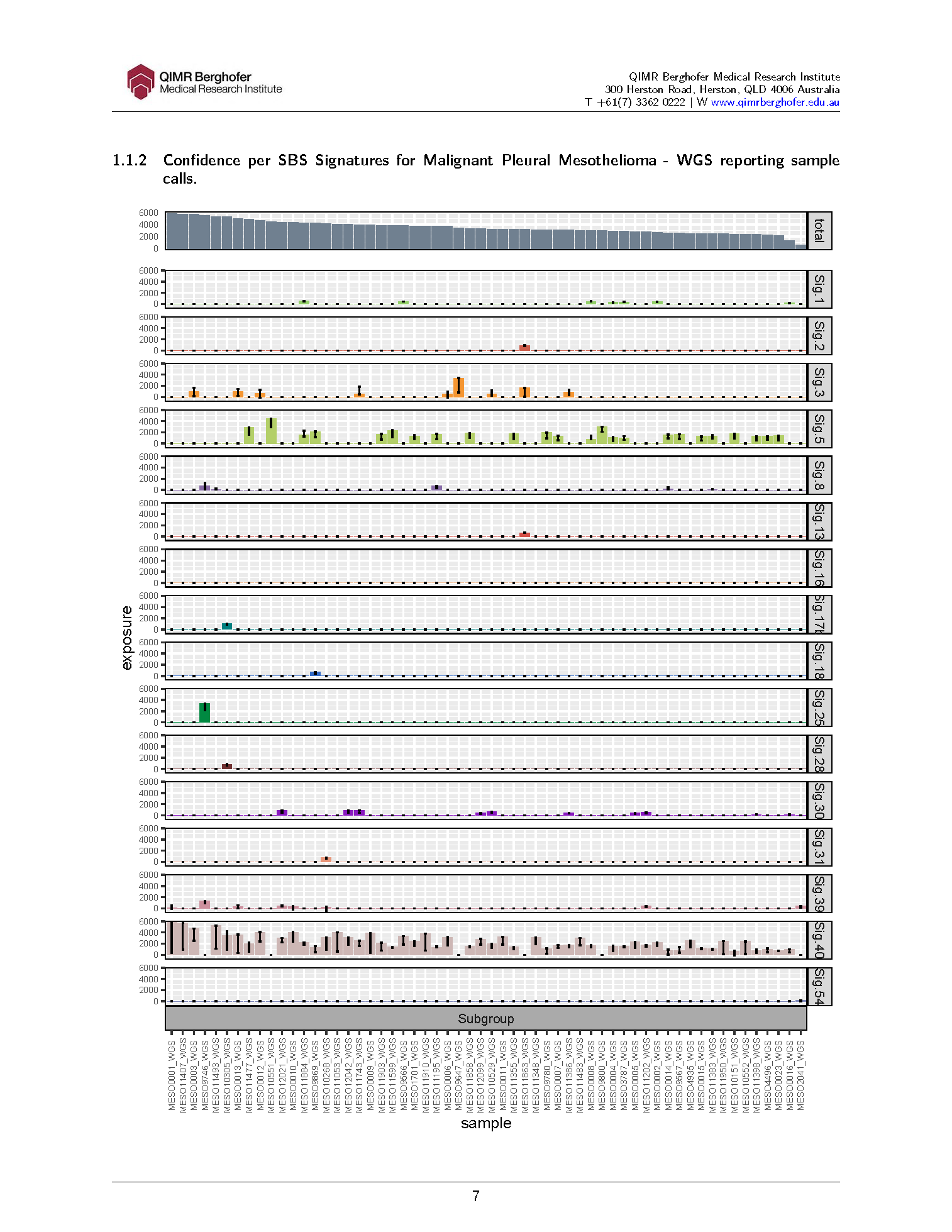
**

**Fig. S2. Single base substitution somatic mutational signature identified in MPM.** The 16 SBS mutational signatures identified by YAPSA in 58 mesothelioma samples sequenced by whole genome sequencing. The upper plot is the total number of somatic SNVs per patient, with patients ordered by highest to lowest number of mutations. The remaining plots show the number of somatic SNVs (y axis labelled as exposure) contributing to each signature per patient (x axis). The SBS signatures are labelled with the COSMIC V3 signature number. The black error bars are the 95% confidence interval for each signature within each sample.

**Fig. S3. Somatic short indel mutational signatures identified in MPM.** The 11 ID mutational signatures identified by YAPSA in 58 mesothelioma samples sequenced by whole genome sequencing. The upper plot is the total number of somatic indels per patient, with patients ordered as in Fig. S1. The remaining plots show the number of somatic indels (y axis labelled as exposure) contributing to each signature per patient (x axis). The ID signatures are labelled with the COSMIC V3 signature number. The black error bars are the 95% confidence interval for each signature within each sample.


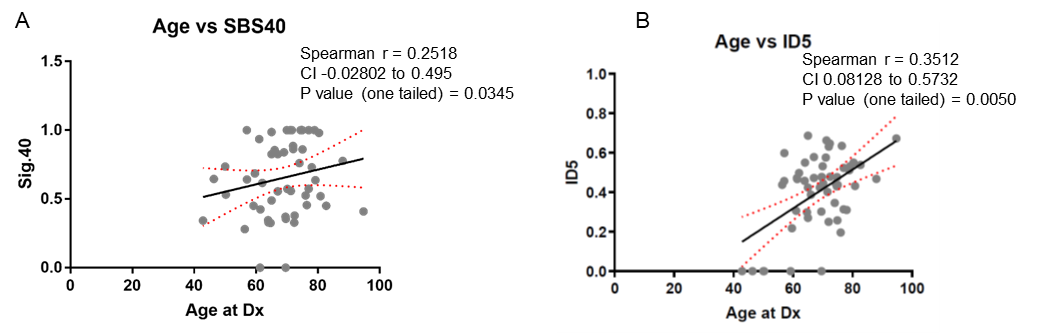


**Fig. S4. Spearman correlation of age of diagnosis with mutational signatures.** A) Age of diagnosis (x-axis) correlated with the proportion of the SBS40 signature (y-axis) for each patient. B) Age of diagnosis (x-axis) correlated with the proportion of the ID5 signature (y-axis) for each patient.

**Fig. S5. Gene expression of MPM genes in tumours with and without mutation in the Creaney at al. cohort.** The expression of the 13 MPM associated genes from Figure 3 is shown. Samples are grouped by sample type (cell line, pleura and effusion), then by whether the gene contains a mutation (Mutated, blue) or no mutation (Wildtype, red). Cases are consider mutated if they contain a SNV, indel, SV, homozygous loss or high gain in the gene of interest. Promoter mutations are also included for the *TERT* gene. *P*-values shown are from Wilcox test.

**Fig. S6. Gene expression of MPM genes in tumours with and without mutation in TCGA cohort.** The expression of the 12 MPM associated genes from Figure 3 is shown. The *TERT* gene is not plotted as the TCGA data was whole exome and therefore *TERT* promoter mutations could not be identified. Samples are grouped by whether the gene contains a mutation (Mutated, blue) or no mutation (Wildtype, red). This is exome sequence data, therefore cases are consider mutated if they contain a SNV or indel in the gene of interest. *P*-values shown are from Wilcox test.

**Fig. S7. Indel neoantigen load in MPM.** A) Predicted total neoantigen load derived from indels with IC50 ≤ 500nM in Creaney et al. cell line, pleura and pleural effusion samples and TCGA pleura samples. B) Percent of neaoantigens from indels that are expressed in Creaney et al. cell line, pleura and pleural effusion samples and TCGA pleura samples. Strong binding neoantigens (IC50≤50nM) shown in blue, weak binding neoantigens (IC50≤50-500nM) shown in yellow and neoantigens that are not detected in RNA-seq are show in black. C) Survival plot of indel neoantigen load in Creaney et al. D) Survival plot of indel neoantigen load in TCGA samples. Survival plots were classified based on top and lower quartiles of neoantigen load of both datasets.


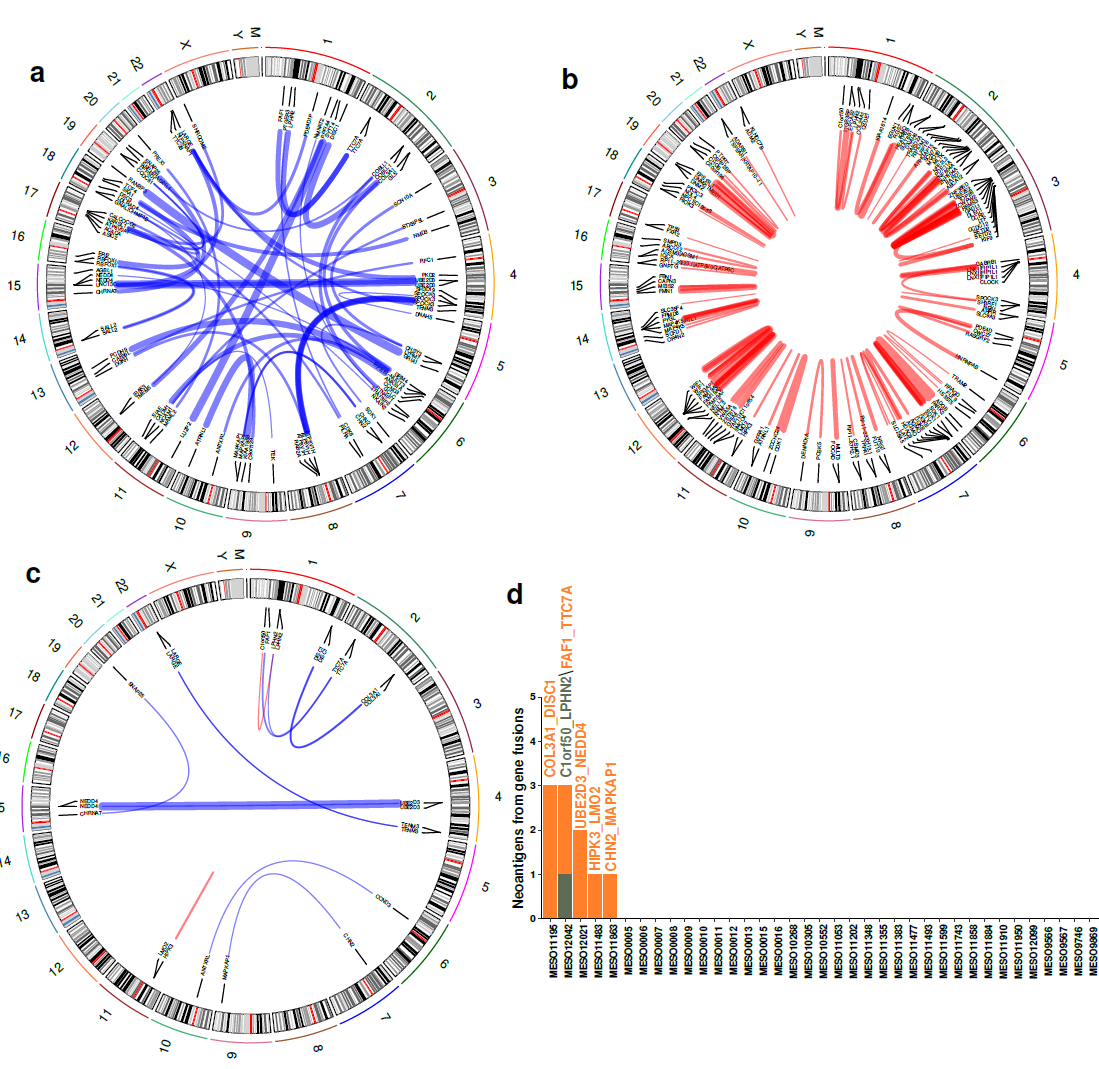


**Fig. S8. Gene fusion events and neoantigen prediction in MPM.** A) Circos plot showing candidate gene fusion events caused by inter-chromosomal rearrangements identified by WGS. B) Circos plot showing candidate gene fusion events caused by intra-chromosomal rearrangements identified by WGS. C) Circos plot showing 13 gene fusion events identified by WGS and confirmed in RNA-Seq. D) The number of neoantigens which are predicted from each gene fusion event per patient.


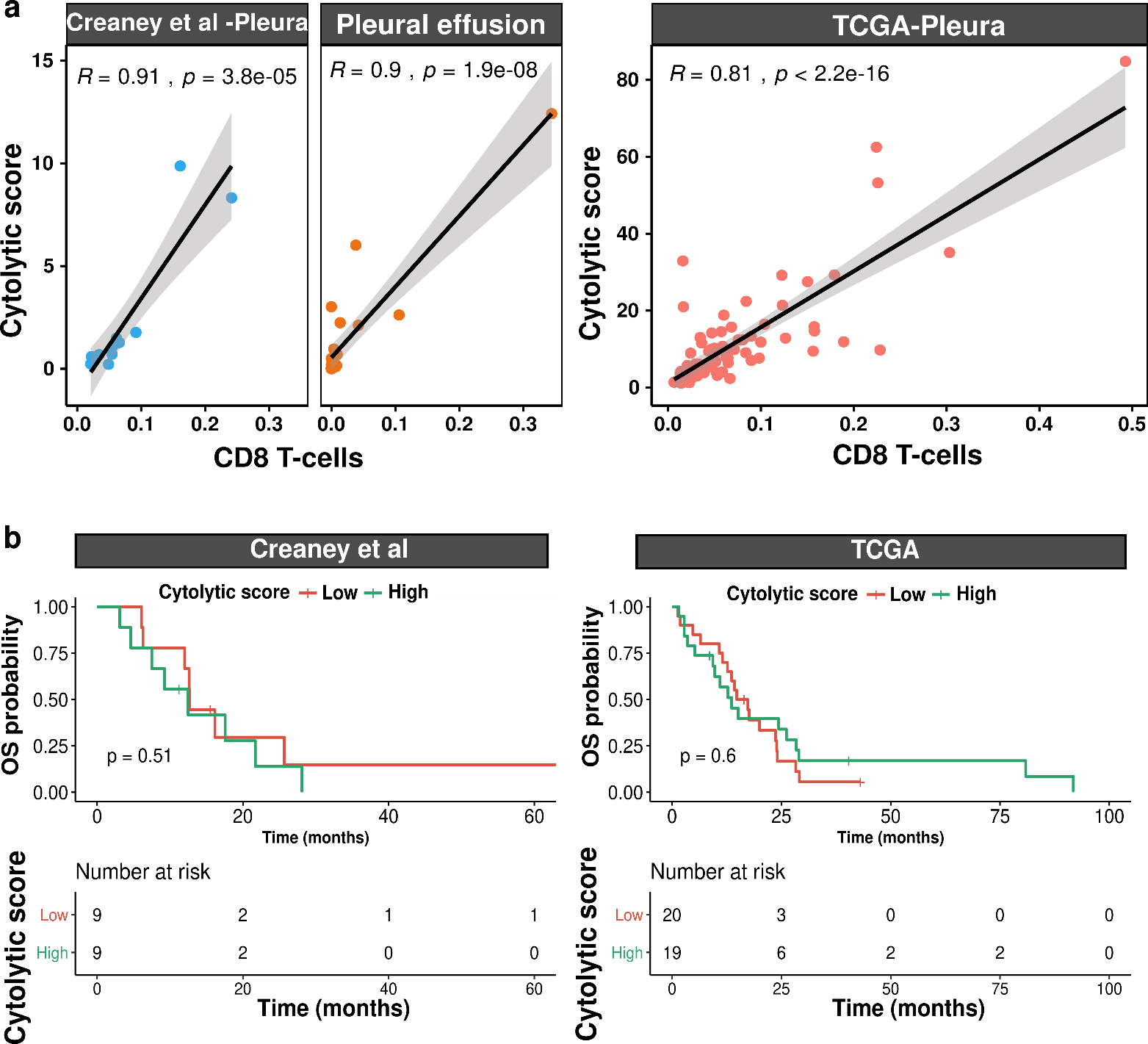


**Fig. S9. Cytolytic activity and survival in MPM.** A) Cytolytic score (estimated from mRNA expression levels of *GZMA* and *PRF1*) showing significant positive correlation (pearson) with the proportion of CD8 T-cells estimated with CIBERSORT. B) Kaplan-Meier plots of samples showing no association with overall survival in patients with high (top quartile) cytolytic score and low (bottom quartile) in Creaney et al. (left panel) and TCGA cohorts (right panel).

**Fig. S10. Chemokine, cytokine, interleukin and matrix metalloproteases expression in MPM.** The expression of chemokine, cytokine, interleukin and matrix metalloproteases in A) Creaney et al. and B) TCGA samples**.** Samples are on the x-axis and log-transformed gene expression values on the y-axis. Samples and gene expression values were clustered using a non-supervised hierarchical clustering. C) Kaplan-Meier plots of *CCL2*, *MMP2* and *MMP14* gene expression values in Creaney et al. (left panels) and TCGA datasets (right panels). Samples are divided into lower, middle and upper tertile based on expression.


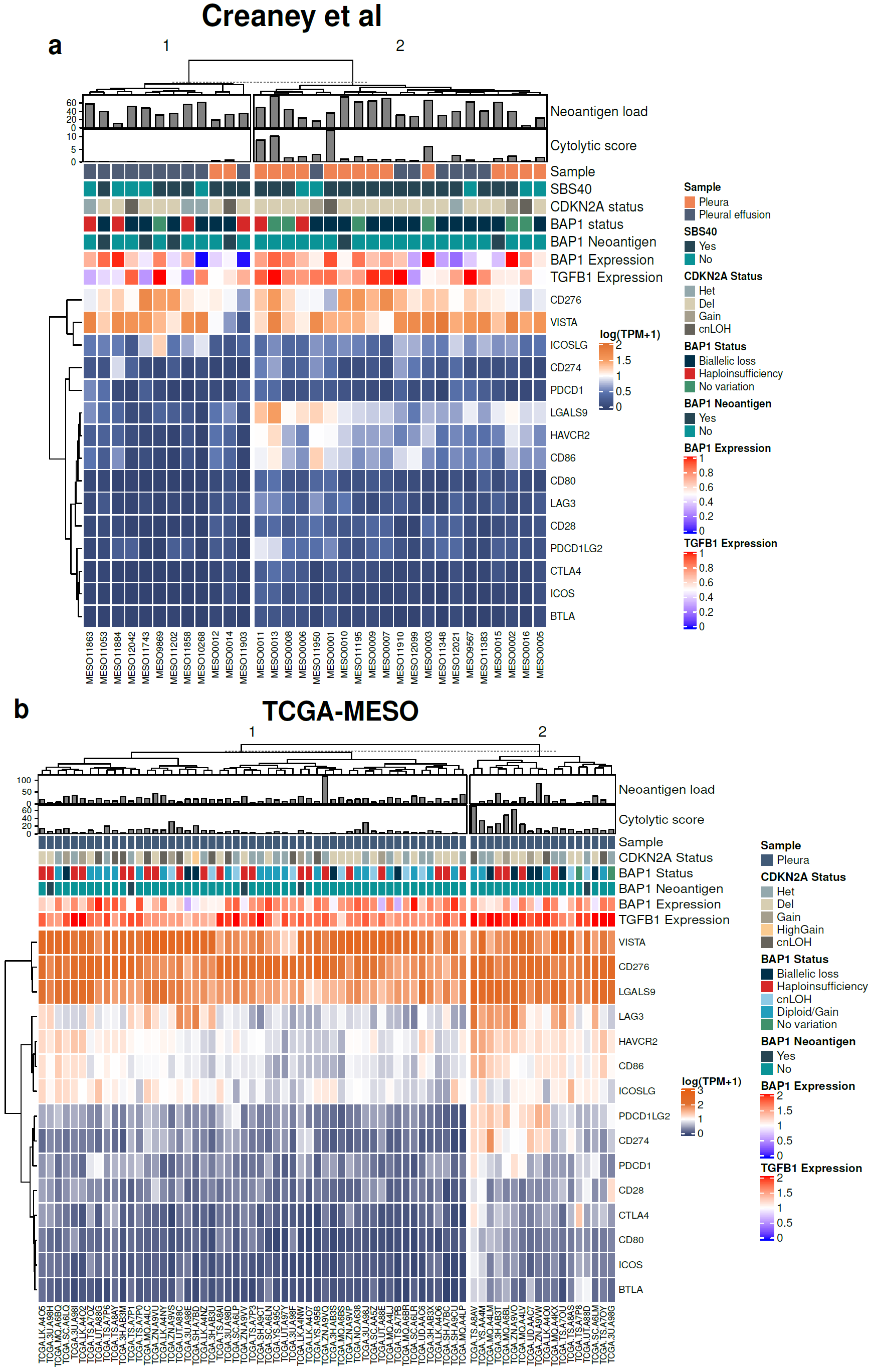


**Fig. S11. Expression of immune checkpoint receptors in MPM.** The expression of immune checkpoint receptors in A) Creaney et al. and B) TCGA samples. Samples are on the x-axis and log-transformed gene expression values on the y-axis. Samples and gene expression values were clustered using a non-supervised hierarchical clustering. The neoantigen load (IC50<500nM) from SNVs and indels is shown in the upper bar plot, cytolytic score estimated by geometric mean of *GZMA* and *PRF1* gene expression are in the lower bar plot. In the colour bar the *CDKN2A* mutation status, *BAP1* mutation status and whether the *BAP1* mutations (SNVs, frameshift of indels) are predicted to form neaoantigens that may illicit immunogenic response is shown. *TGFB1* expression in low, mid and high tertiles is shown in the colour bar.
